# The Relationship between Cancer Stage, Selected Immunological Parameters, Epstein–Barr Virus Infection, and Total Serum Content of Iron, Zinc, and Copper in Patients with Laryngeal Cancer

**DOI:** 10.3390/jcm13020511

**Published:** 2024-01-16

**Authors:** Julia Wojnicka, Ewelina Grywalska, Anna Hymos, Paulina Mertowska, Sebastian Mertowski, Małgorzata Charytanowicz, Maria Klatka, Janusz Klatka, Wojciech Remington Dolliver, Anna Błażewicz

**Affiliations:** 1 Department of Pathobiochemistry and Interdisciplinary Applications of Ion Chromatography, Medical University of Lublin, 1 Chodźki Street, 20-093 Lublin, Poland; julia.wojnicka@umlub.pl; 2 Department of Experimental Immunology, Medical University of Lublin, 4a Chodźki Street, 20-093 Lublin, Poland; ewelina.grywalska@umlub.pl (E.G.); anna.hymos@umlub.pl (A.H.); paulina.mertowska@umlub.pl (P.M.); sebastian.mertowski@umlub.pl (S.M.); 3 Department of Computer Science, Faculty of Electrical Engineering and Computer Science, Lublin University of Technology, Nadbystrzycka 38D, 20-618 Lublin, Poland; m.charytanowicz@pollub.pl; 4 Systems Research Institute, Polish Academy of Sciences, Newelska 6, 01-447 Warsaw, Poland; 5 Department of Pediatric Endocrinology and Diabetology, Medical University, Gębali 1 St., 20-093 Lublin, Poland; maria.klatka@umlub.pl; 6 Department of Otolaryngology and Laryngological Oncology, Medical University of Lublin, Jaczewskiego 8 St., 20-954 Lublin, Poland; janusz.klatka@umlub.pl; 7 Brigham & Women’s Hospital, 75 Francis St., Boston, MA 02115, USA; wdolliver@bwh.harvard.edu

**Keywords:** iron, zinc, copper, immunophenotype, EBV, laryngeal cancer

## Abstract

(1) Background: the purpose of the study was to assess the relationship between cancer stage, selected immunological parameters, Epstein–Barr virus (EBV) infection, and total serum content of iron, zinc, and copper in patients with laryngeal cancer (LC). (2) Methods: serum Fe, Zn, and Cu were measured in 40 LC patients and 20 controls. Immunophenotyping of peripheral blood lymphocytes was performed by flow cytometry using fluorescent antibodies against CD3, CD4, CD8, CD19, CD25, CD69, and PD-1. Tumor and lymph node lymphocytes were analyzed by flow cytometry. EBV DNA was quantified by real-time PCR, targeting the *EBNA-1* gene. Associations between serum elements, immune markers, and cancer grade/stage were evaluated using ANOVA and appropriate nonparametric tests. (3) Results: levels of Fe, Cu, and Zn were lower, while Cu/Zn was statistically higher, in patients with LC than in the control group. Correlation analysis showed a statistically significant association between the levels of these elements and parameters of the TNM (Tumor, Node, Metastasis) staging system, immunophenotype, and the amount of EBV genetic material in patients with LC who survived for more than 5 years. (4) Conclusion: the results suggest that the total serum levels of the determined micronutrients may significantly affect the immunopathogenesis and progression of LC.

## 1. Introduction

According to 2020 data available from The International Agency for Research on Cancer (WHO), laryngeal cancer is the third most common cancer affecting the face and neck region [1]. Laryngeal cancer in its early stages may not have noticeable symptoms, leading to delayed diagnosis. As the disease progresses, typical symptoms may include persistent hoarseness, difficulty breathing or swallowing, ear pain, persistent coughing and unexplained weight loss. It affects people in various demographic groups and regions, over the age of 40, and is more common among men than women [2,3]. The risk of laryngeal cancer is mainly due to damage to the cells lining the larynx, or damage to the vocal cords, caused by factors such as alcohol, tobacco, environmental pollutants, disease (laryngitis, gastroesophageal reflux disease), or viral infections such as human papilloma virus (HPV) and Epstein–Barr virus (EBV) [4]. Our recently published study revealed that EBV infection may affect the PD-1/PD-L1 pathway and the development of laryngeal cancer; moreover, the level of PD-1 on CD4+ T cells in lymph nodes may serve as a marker for laryngeal cancer treatment and prognosis [5]. EBV is oncogenic to two cell types: B lymphocytes and epithelial cells. As a result of the lytic cycle and latent overproduction of B cells, there is an increased risk of lymphoma formation. Lactoferrin, an iron-binding glycoprotein, may play a role in the inhibition of EBV infection, as it was shown in Zheng Y. et al. studies [6]. Lactoferrin has been shown to not only inhibit cytomegalovirus and HPV infections, but it can also be an inhibitor in the infection of B lymphocytes by EBV, as well as prevent virus transfer from lymphocytes to epithelial cells by interfering with tumor cell growth. 

Toxic metals such as cadmium, lead and arsenic are known to be carcinogenic and have a significant effect on the development of cancers of the larynx, lung, and nose [7,8,9,10]. However, it is unknown whether the excess accumulation of essential metals that are under tight homeostatic control in the body, such as iron, may induce the production of highly reactive hydroxyl radical species and have a role in cancer pathogenesis. Our attention has been focused on essential trace elements: iron, zinc and copper, which play a key role in various physiological processes in the body, particularly the functioning and the effectiveness of the immune system, which is extremely important for defense against cancer. Fe, Zn and Cu are essential components of various enzymes and proteins involved in immune system function, cell growth and DNA repair. Disturbances in the concentrations of these elements can interfere with the precise mechanisms that keep the immune system alert, potentially tipping the scales in favor of tumorigenesis. Concentrations of Fe, Zn and Cu in body fluids are maintained within certain ranges, with their deficiencies being more common than their toxic effects after overdose [11,12,13,14]. The level of micronutrients in the human body is profoundly influenced by nutritional status, representing a critical factor in maintaining overall health. An aberrant nutritional state, marked by either a deficiency or excess of these micronutrients, can give rise to significant health implications. This impact transcends the prevention of infections; it extends to the modulation of disease severity and the exacerbation of pre-existing health issues. The repercussions of abnormal nutritional levels, whether due to deficiency or, less commonly, excess, are far-reaching. Such deviations can lead to impaired normal development, sustained maintenance, and the effective expression of immune functions essential for a robust defense system. This, in turn, heightens the risk of infection and exacerbates the potential for cancer development. The reduction in the number and activity of crucial immune cells, including lymphocytes, underscores the intricate relationship between nutritional status and immune competence [15].

Copper, along with zinc, plays a role in cell growth, antioxidant defense and the function of enzymes involved in energy production. They act as cofactors and maintain the activity of the vitally important enzyme superoxide dismutase (SOD), helping to neutralize harmful reactive oxygen species (ROS) produced during immune responses that lead to cell and tissue damage [16,17]. Copper is essential for the development and function of neutrophils, as well as during the maturation of B and T lymphocytes [18]. In turn, zinc is involved in the development and activation of T lymphocytes, B lymphocytes and NK cells [19,20,21]. Zinc also plays a role in chemotaxis and is involved in regulating the production of pro-inflammatory and anti-inflammatory cytokines [22,23]. Elemental deficiency can impair the ability of immune cells, such as neutrophils, causing them to migrate to sites of infection, engulf pathogens and generate ROS. Elemental deficiency may also disrupt the balance of cytokines, potentially impairing immune system regulation and response to infection. Copper is also involved in regulating the metabolism of other elements, such as iron. It is an important component of the enzyme ceruloplasmin, which is produced in the liver and is essential for the conversion of ferrous iron (Fe^2+^) into the more easily transportable form of ferrous iron (Fe^3+^) in the bloodstream. This conversion is crucial for the binding of iron to transferrin, a transport protein that carries iron through the bloodstream to various tissues. In essence, ceruloplasmin acts as a ferroxidase, facilitating the oxidation of iron for proper transport [24]. Moreover, the metal affects the stability of ferroportin, a trans-membrane protein found on the surface of small intestinal cells, macrophages and other tissues, responsible for the export of iron from cells into the bloodstream [25]. It also participates in regulating the expression of hepcidin, a peptide hormone produced by the liver that regulates iron homeostasis. Copper deficiency can lead to hepcidin dysregulation, potentially affecting iron absorption and distribution, as well as impairing the absorption of nonheme iron, the type of iron found in plant-based foods and supplements [26].

Iron serves as a carrier of oxygen to the tissues from the lungs by hemoglobin, but it is involved in many other important cellular processes. Besides cellular respiration (in which iron forms, among other things, the last protein in the respiratory chain—cytochrome c oxidase), this element plays a role in energy metabolism by being a component of succinate and isocitrate dehydrogenases. Fe is the component of enzymes, e.g., cytochrome P450, important in the synthesis of steroid hormones. Fe is involved in DNA replication and synthesis, nucleic acid repair, heme and iron-sulfur cluster (ISC) synthesis, as well as being a hydroxylase or oxidoreductase in signaling [27,28,29,30]. In some situations, nevertheless, this micronutrient can change from beneficial to potentially toxic. This occurs when ferrous iron donates an electron to hydrogen peroxide, going from the Fe^2+^ to Fe^3+^ state, followed by iron regeneration (referred to as the Fenton cycle) [31]. The result of these reactions is the generation of Fe^2+^ and free radical-reactive oxygen species (ROS), specifically hydroxyl radicals [32,33]. These molecules, acting as the most important oxidants in the human body, can unfortunately lead to peroxidation and apoptosis, i.e., programmed cell death, targeting the most important building particles such as carbohydrates, nucleic acids, lipids, and proteins [34]. Elements of the immune system are also involved: programmed death 1 receptor (PD-1), programmed death-ligand 1 (PD-L1), and cytotoxic T cell antigen 4 (CTLA 4). The regulation of iron emerges as a crucial element in the sophisticated antimicrobial mechanisms employed especially by monocyte-derived cells and macrophages. These forager cells have honed strategies, termed ‘nutritional immunity,’ to intricately control the availability of iron to microorganisms. Through sequestration within intracellular compartments, macrophages create a less accessible environment for pathogens and potential cancer cells. This reduction in iron availability, a pivotal component of the host’s enzymes, serves to create an unfavorable milieu, hampering the replication of viruses. Macrophages exhibit a remarkable ability to regulate iron homeostasis, enlisting the assistance of key players such as nitric oxide (NO) and iron regulatory proteins (IRPs) like lactoferrin, transferrin, and lipocalin. This allows macrophages to finely control the accessible levels of iron for microorganisms, playing a pivotal role in the host’s defense mechanisms [35]. Zinc deficiency may also have a considerable effect on modulating immune function, particularly in the context of macrophage activity and cytokine regulation as a result of the abnormal action of zinc-dependent endopeptidases such as extracellular matrix metalloproteinases (MMPs) [36]. Beyond the realm of microbial threats, the significance of iron regulation takes on added complexity. Unchecked iron levels can lead to amplified oxidative stress, potentially triggering the activation of precancerous pathways and providing support for the growth of tumor cells [37]. In the progression of not only laryngeal cancer but many other types of cancer, a form of free iron not bound to ferritin, called labile iron, plays an essential role [38,39]. Excessively accumulated labile iron is prone to engage in the catalytic generation of ROS. Free radicals formed in reactions catalyzed by iron can oxidize lipids, proteins, lipoproteins and nucleic acids, leading to cell death and tissue damage, or even to cell phenotype change, tumor formation and cancer [38]. In the available literature, there are no conclusive studies indicating that excess iron may be one of the carcinogenic factors [40,41,42,43,44,45,46,47]. Iron, by causing oxidative stress, can modify the genome, causing changes in tumor morphology and increasing the possibility of metastasis [48]. In the case of hepatocellular carcinoma, the excessive absorption of iron from the gastrointestinal tract, causing hemochromatosis, results in abnormal gene hypermethylation, which may mean that iron-induced epigenetic modification can initiate malignant transformation [49]. In addition, this micronutrient, by modifying the extracellular matrix, activating promigratory signaling pathways and suppressing the immune response, can transform the tumor area and increase the risk of metastasis [38].

Taking into consideration the above information and the results of our previous studies, as well as the availability of biological material collected in the project, we hypothesized that changes in serum levels of iron, zinc and copper would correlate with immunophenotypic changes and disease severity in patients with laryngeal cancer. This study aimed to assess relationships between serum levels of these elements, immune parameters, and cancer stage/grade in laryngeal cancer patients compared to controls. Studies of essential metals may be useful for the biological monitoring of deficiencies during disease states. The study of this trio of essential metals that influence the processes related to inflammation in the body and the development of cancer in the case of their deficiency allows for a broader insight into the processes occurring during the disease than would be the case when studying individual metals. Moreover, we determined the effect of total serum iron, zinc and copper content on immune function and the reactivation of EBV, a risk factor for the incidence of this type of cancer. Due to the important role of the immune system in the pathogenesis of laryngeal cancer, selected parameters of the immunophenotype of patients with laryngeal cancer and the control group were also analyzed.

## 2. Materials and Methods

### 2.1. Patient Characteristics

**Figure 1:**
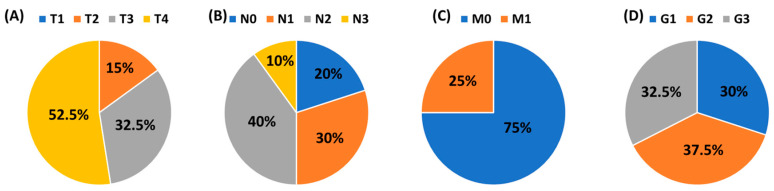
The clinical and histological stage of laryngeal cancer patients according to the TNM system and the histological grade (G1–G3). (**A**) T: size or direct extent of the primary tumor; (**B**) N: degree of spread to regional lymph nodes; (**C**) M: presence of distant metastasis; (**D**) Grade.

This study was conducted in accordance with the guidelines of the Helsinki Declaration and approved by the Ethics Committee of the Medical University of Lublin (KE-0254/70/2015. approval date: 26 March 2015). The research was carried out on 40 patients diagnosed with squamous cell carcinoma of the larynx (LSCC), who were qualified by the Department of Otolaryngology and Oncology, Medical University of Lublin. The mean age of 40 men was 62.17 ± 6.55 years. The health condition of each patient was confirmed histologically. The control group included 20 volunteers hospitalized with a deformation of the nasal septum, whose mean age was 64.40 ± 8.88 years. The clinical and histological stage of laryngeal cancer patients was classified according to the TNM system (Tumor, Node, Metastasis) and histological grade (G1–G3). A detailed distribution of patients is presented in Figure 1. Patients with advanced head and neck tumors required advanced, multi-hour surgery. The study group in Figure 1 does not contain patients in the T1 stage since they are treated at other centers that do not belong to the university.

The patients diagnosed with cancer did not suffer from underlying illnesses other than laryngeal cancer. Laryngeal cancer patients and the control group did not show human immunodeficiency virus, hepatitis B virus, hepatitis C infection or allergic diseases. All the participants in this study were not taking any medications that could affect the immune system, nor had they had any recent blood transfusions. The patients were not on any special diet prior to sampling. The blood used for the study was drawn in the morning, due to daily changes in element metabolism, from fasting patients during a routine medical visit. After analyzing the basic parameters of peripheral blood morphology, it was found to be within the accepted reference ranges. Determined parameters include: WBC, White Blood Cells [4.0–10.0 × 10^3^/mm^3^]; NEU, neutrophils [1.8–7.5 × 10^3^/mm^3^]; LYM, lymphocytes [0.6–3.4 × 10^3^/mm^3^]; MON, monocytes [0.0–0.9 × 10^3^/mm^3^]; EOS, eosinophils [0.0–0.7 × 10^3^/mm^3^]; BAS, basophils [0.0–0.2 × 10^3^/mm^3^]; RBCs, red blood cells [4.5–6.5 × 10^6^/mm^3^]; HGB, hemoglobin [13.0–17.5 g/dL]; HCT, hematocrit [40–54%]; MCV, mean corpuscular volume [77–93 fL]; MCH, mean corpuscular hemoglobin [27–32 pg]; MCHC, mean corpuscular hemoglobin concentration [32–38 g/dL]; RDW, red blood cell distribution width [10.0–15.0%]; PLT, platelets [150–400 × 10^3^/mm^3^]; ferritin [15–400 µg/L]; and transferrin [2–4 g/L]. Study participants did not suffer from hemochromatosis and did not take metal-chelating drugs or dietary supplements containing iron, zinc or copper. No patients were found to be malnourished after being interviewed by a dietician. Both transferrin and hemoglobin, as measured by commercially available tests, were within normal ranges for all study participants.

### 2.2. Flow Cytometric Analysis of Cells in Peripheral Blood

**Figure 2:**
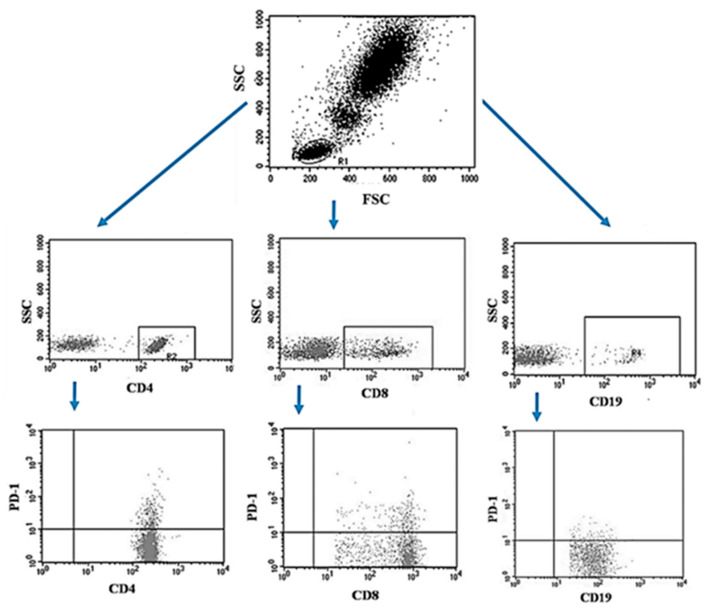
Example of the flow cytometric analysis of CD4+, CD8+, CD19+ PD-1+ lymphocytes in a peripheral blood sample.

The peripheral blood of the patients was collected using the S-Monovette^®^ system into tubes containing EDTA (SARSTEDT AG & Co. KG Sarstedtstraße, Nümbrecht Germany), then, the collected blood was immediately transported to the laboratory under the conditions of 4–6 °C. Preparation of the sample for analysis included a 30-min incubation period (in the dark, 4 °C) of blood with labeled monoclonal antibodies directed at the following differentiation antigens: CD3 (FITC), CD19 (PE), CD4 (FITC), CD8 (PE), CD69 (PE-cy5), CD25 (PE-cy5), CD8 (FITC), CD19 (FITC), PD-1 (PE) (Franklin Lakes, BD, USA). After the incubation, the test sample was exposed to the lysing solution to get rid of erythrocytes, according to the manufacturer’s instructions (Franklin Lakes, BD, USA). Then the samples were washed twice with phosphate buffered saline (PBS) without Ca^2+^ or Mg^2+^ (Sigma-Aldrich, Merck, Germany). The sample prepared in this way was analyzed on a FACSCalibur flow cytometer (Franklin Lakes, BD, USA), for each sample 10,000 events were collected [5]. An example of the flow cytometric analysis of CD4+, CD8+, CD19+ PD-1+ lymphocytes in peripheral blood sample is shown in Figure 2.

### 2.3. Flow Cytometric of Cells in Tumor Tissue and Lymph Nodes

**Figure 3:**
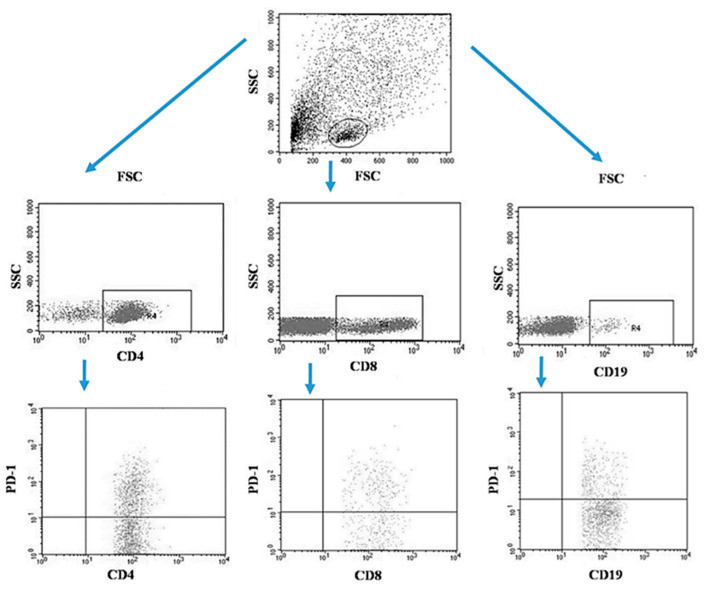
Example of the flow cytometric analysis of CD4+, CD8+, CD19+ PD-1+ lymphocytes in the tumor sample.

**Figure 4:**
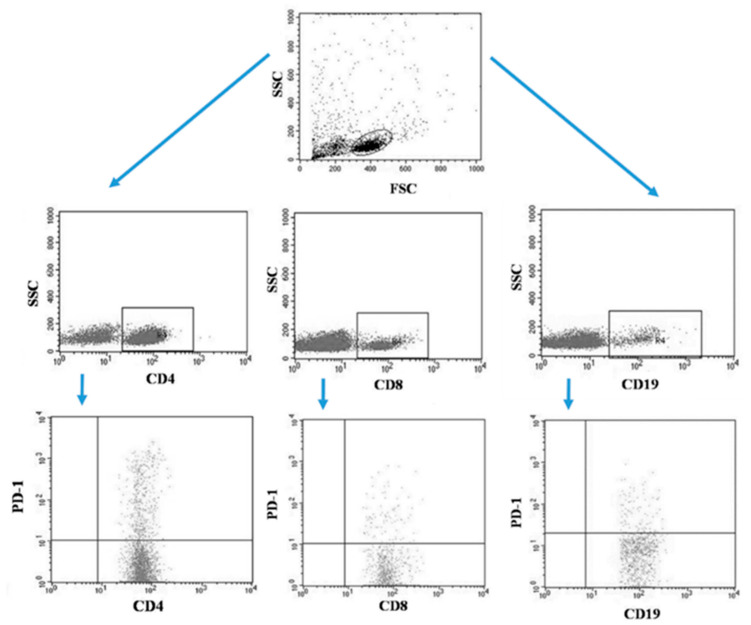
Example of the flow cytometric analysis of CD4+, CD8+, CD19+ PD-1+ lymphocytes in the lymph node sample.

Tumor samples and patients’ lymph nodes were collected during surgical treatment and then transported under appropriate conditions to the laboratory where they were digested with 1 mg/mL type I collagenase (Sigma-Aldrich, Merck, Germany). The resulting cell suspensions were filtered through a 40 µm nylon cell strainer (Falcon; Becton Dickinson, East Rutherfort, NJ, USA). The filtered cell suspension was the starting material for the isolation of mononuclear cells by the density gradient method, using the Gradisol-L reagent (Aqua Medica, Poland). The obtained mononuclear cells were washed twice with phosphate-buffered saline (PBS) solution free of Ca^2+^ or Mg^2+^ (Sigma-Aldrich, Merck, Germany). Peripheral blood mononuclear cells (PBMCs) were suspended in PBS at a density of 2 × 10^6^ cells/mL. The viability of mononuclear cells after tissue digestion was verified by microscopy methods using Trypan blue (0.4% trypan blue solution; Sigma-Aldrich, Hamburg, Germany). The obtained PBMCs were incubated for 30 min in the dark at 4 °C with the following labelled monoclonal antibodies targeting the following differentiation antigens: CD3 (FITC), CD19 (PE), CD4 (FITC), CD8 (PE), CD69 (PE-cy5), CD25 (PE-cy5), CD8 (FITC), CD19 (FITC), PD-1 (PE) (Franklin Lakes, BD, USA). After the incubation period, cells were washed twice with PBS. The samples prepared in this way underwent the same cytometric analysis as the blood samples [5]. Figure 3 illustrates an example of the flow cytometric analysis of CD4+, CD8+, CD19+ PD-1+ lymphocytes in the tumor sample, whereas Figure 4 refers to the lymph node sample.

### 2.4. Analysis of the Total (Organic and Inorganic) Content of Fe Zn, Cu, and Cu/Zn in Serum

Blood samples for the determination of Fe, Zn, Cu, and Cu/Zn content were collected after overnight fasting and immediately transferred to non-heparinized labeled serum separator tubes. These tubes were then centrifuged at 5000× *g* rpm for 10 min to obtain serum and stored in PTFE containers below −20 °C until ion chromatography (IC) analysis. To evaluate total serum Fe, Zn, and Cu content (the mineralization procedure of the samples makes both organic and inorganic iron forms converted to Fe^3+^; total serum Cu is determined as Cu^2+^; and total serum Zn as Zn^2+^), each time, 1 mL of serum underwent the digestion procedure described by the authors [50], and samples were mineralized using a microwave-assisted high-pressure digestion system. The digestion was carried out in the NovaWAVE Microwave Tunnel Digestion System (SCP Science, Canada) using Teflon^®^ vessels. The microwave-assisted sample preparation was conducted in a closed system. Each time, an acidic digestion with 65% nitric acid water solution was applied (1 mL of HNO_3_: 9 mL of deionized H_2_O).

Ion chromatography (IC) analyses were performed on a Dionex DX500 ion chromato-graph (Dionex Co., Sunnyvale, CA, USA) equipped with an IP 25 isocratic pump, a 25 mL injection loop, a PC 10 Pneumatic Controller post-column reactor, an AD20 (UV/Vis) detector, and a Chromeleon chromatography workstation for instrument control and data acquisition. The detailed methodology of IC and the validation procedures are described elsewhere [51].

### 2.5. Assessment of the Presence of Specific Antibodies EBV IgM VCA, EBV IgG VCA

The concentration of the specific antibodies EBV IgM VCA and EBV IgG VCA were assessed in the plasma of the test and control groups using commercial ELISA kits (Demeditec, Germany, with a limit of detection of 10 U/mL). The analysis was prepared according to the manufacturer’s instructions and the results were read at a wavelength of 450 nm using a Victor TM3 instrument (PerkinElmer, Waltham, MA, USA). The antibody concentration was read from the standard curve [5].

### 2.6. Assessment of the Amount of Viral DNA in the Studied Patients

Patient DNA was isolated from one million PBMCs using the QIAamp DNA Blood Mini Kit (QIAGEN Strasse 1, 40724 Hilden, Germany) according to the kit instructions. Subsequently, the concentration of the isolated material was assessed using a BioSpecnano spectrophotometer (Shimadzu, Kyoto, Japan). The EBV DNA copy number was assessed using the ISEX variant of the EBV PCR kit (GeneProof, Brno, Czech Republic). The samples were analyzed in duplicate with DNA elution buffer as an additional negative control. A specific conserved DNA sequence for the EBV nuclear antigen 1 gene (*EBNA-1*) was amplified using a 7300 Real-Time PCR System (Applied Biosystems, Waltham, MA, USA). Due to the detection limit of the assay of ten EBV DNA copies/μL, all samples below this threshold were considered EBV-negative [5]. 

### 2.7. Statistical Analysis

Statistical analyses were performed using Statistica 13 (TIBCO Software Inc., Palo Alto, USA). The continuous experimental data were expressed as mean ± SD, median, and range. Discrete data were tabulated as numbers and percentages. The Student’s *t*-test for independent samples was used as the inference test according to assumptions of normal distribution, and this was checked with the Shapiro–Wilk test and the Kolmogorov–Smirnov test (*p* < 0.05). Homogeneity of variance was checked with Levene’s test (*p* < 0.05). If the assumption of homogeneity of variance was not fulfilled, an unequal variances *t*-test was applied. If the assumption of normal distribution was not fulfilled, the Mann–Whitney U test was applied. The differences between more than two groups were analyzed by ANOVA or an ANOVA Kruskal–Wallis test with post hoc multiple comparison tests (*p* < 0.05). Spearman’s correlation coefficient and corresponding significance test were applied to examine the relationship between two variables (*p* < 0.05).

## 3. Results

### 3.1. Characteristics of Selected Parameters of Peripheral Blood Count, Serum Iron, Zinc and Copper Concentration and Immunophenotype in Patients with Laryngeal Cancer and in the Control Group

A detailed analysis of selected blood count parameters and serum iron, zinc and copper concentration for patients diagnosed with laryngeal cancer and for patients from the control group is presented in Table 1.

Serum Fe level reflects the amount of circulating iron bound to transferrin, and in healthy men, it is in the range of 80–180 mcg/dL [52]. In our study, the average serum Fe level in patients with laryngeal cancer was significantly decreased compared to cancer patients in the control group (mean 79.87 vs. 100.86 mcg/dL, *p* = 0.0005). We observed a statistically significant decrease in hemoglobin and hematocrit values in relation to patients from the control group and an increase in the RDW value in those patients, which was accompanied by a slight decrease in MCV, which occurs in the case of iron deficiency in the body. Changes in the levels of zinc and copper and the copper/zinc ratio were also statistically significant. Patients with laryngeal cancer had lower levels of zinc and copper (Zn: 66.36 vs. 83.89 mcg/dL, *p* < 0.00001; Cu: 78.24 vs. 81.96 mcg/dL, *p* = 0.0476). The Cu/Zn ratio was elevated in cancer patients versus controls (1.23 vs 0.98, *p* = 0.00001).

The frequency of PD1-positive T and B lymphocytes was analyzed in detail, which, as we have shown earlier, may be one of the prognostic factors for laryngeal cancer.

Immunophenotype results were performed on both peripheral blood as well as tumors and lymph nodes (Table 2). Frequencies of subpopulations within the leukocyte (CD45+) population are shown.

Peripheral blood immunophenotyping showed significantly increased CD69+ and PD-1+ T cells in cancer patients (*p* < 0.01). The obtained results confirm our previous observations regarding changes in immunophenotype in patients with laryngeal cancer in relation to the control group [5].

### 3.2. Assessment of the Concentration of Selected Elements in Relation to the Grade (G) and TNM Scale

Due to the significant differences between the concentrations of the studied elements among the two studied groups, we conducted a detailed analysis to determine whether the level of Fe, Zn, Cu and Cu/Zn may have a significant effect on the advancement of laryngeal cancer. For this purpose, we used the TNM scale (T–Tumor, N–Node, M–Metastasis) and grade (G). As shown in the obtained results of Table 3, the iron and copper levels, as well as the ratio of copper to zinc, are associated with histologic grade and a noticeable reduction in the levels of both iron and copper with each grade. Iron and copper levels declined with increasing tumor grade, with 36% and 18% reductions between G1 and G3, respectively (*p* < 0.05). In addition, a statistically significant change in the development of metastases was observed. The presence of metastases correlated with a 36.48% decrease in serum iron levels of patients diagnosed with laryngeal cancer and a 9.51% decrease in serum copper levels. Patients with metastases (M1) had 36% lower serum iron versus M0 (*p* = 0.03). The iron, zinc, copper levels as well as copper/zinc ratio were not significantly associated with the size of the primary tumor and metastasis to the lymph nodes (Table 3).

### 3.3. Effect of EBV Reactivation on Laryngeal Cancer Development

EBV reactivation can have a significant impact on the immunopathogenesis of laryngeal cancer. Among all recruited patients, 50% were characterized by the reactivation of EBV in serological profiles, as well as the presence of the virus in genetic material. Detailed information is presented in Table 4. We analyzed the basic parameters related to the presence of EBV in the tested biological material. For this purpose, we determined the EBV DNA copy number/µg DNA in the tumor tissue (mean 266.82 ± 361.23), lymph node (mean 203.69 ± 286.92) and blood (mean 117.96 ± 176.25). Additionally, we quantified the level of Anti-IgM (mean 24.19 ± 8,98) and Anti-IgG VCA EBV (mean 91.52 ± 51.17) (Table 4). All the observed changes were statistically significant between patients diagnosed with laryngeal cancer and patients in the control group (Table 4). The EBV DNA detection threshold was 10 EBV DNA copies/μg; therefore, all samples below this threshold were considered EBV-negative, which is why a result of 0 is included in the control group in Table 4.

In the next step, we conducted a comparative analysis of the various immune system parameters tested, with a special focus on PD-1 expression in EBV+ and EBV- patients (Table 5). In Table 5, we compare patients by EBV reactivation or absence with a group of healthy volunteers who lacked EBV activity. In this table, we present only those parameters that were statistically significant. Meanwhile, the full results are presented in the Supplementary Materials in Table S1. As for the study group, which included laryngeal cancer patients and their division into EBV+ and EBV- patients, statistical significance was observed only in the case of CD19+CD69+ B-lymphocytes in the blood, as well as CD4+PD-1. As for the second comparison, which was EBV+ laryngeal cancer patients and EBV-healthy volunteers, we observed no statistical significance only in the population of CD4+CD25+ lymphocytes in the blood. The last comparison group was patients with EBV-laryngeal cancer versus a group of healthy EBV volunteers, in which we observed a lack of statistical significance only in the population of CD19+CD69+ B lymphocytes, as well as in the IgG VCA EBV quantitative group (Table 5). Our next step was to conduct a similar analysis to the previous one. This time, we wanted to compare the relevance of the selected elements we studied in patients with laryngeal cancer in relation to EBV reactivation and a group of healthy volunteers (Table 6). Detailed analyses of the correlation of individual immune system parameters with the analyzed elements in the context of EBV virus reactivation are presented in Table 7, Table 8, Table 9, Table 10, Table 11 and Table 12.

The total serum iron level (including organic and inorganic iron) significantly positively correlated with the percentage of CD4+ CD3+ lymphocytes T (*p* = 0.020) and the CD4/CD8 ratio (*p* = 0.014). In the immunophenotyping analysis, apart from the basic markers of cellular differentiation CD3+, CD4+, CD8+ and CD19+, the markers CD25+ and CD69+, which are related to the activation of individual subpopulations of lymphocytes, were also used. We have shown that iron levels significantly positively correlated only with the expression of CD4+ CD69+ lymphocytes T (*p* = 0.014) and CD4+ CD25+ T lymphocytes (*p* = 0.023) in tumor samples and CD3+ CD25+ T lymphocytes (*p* = 0.018) in node samples. An analysis of the frequency of occurrence of PD-1-positive on selected lymphocyte subpopulations in the tested biological material in relation to the serum iron level showed a significant negative correlation between T CD8 + PD-1+ lymphocytes (*p* = 0.023) in tumor samples and T CD4+ PD-1+ lymphocytes (*p* = 0.000) in node samples. We did not observe any statistically significant changes between the serum iron level and the analyzed immunological parameters in the case of peripheral blood samples in the studied group of patients. The serum zinc level significantly positively correlated with the percentage of CD19+CD69+ in tumor samples and CD4+CD25+ in node samples. The serum copper level significantly correlated with the percentage of CD3+CD69+, CD4+PD-1 and CD8+PD-1 in tumor samples and CD4+PD-1, CD8+PD-1 in node samples.

### 3.4. The Importance of Serum Iron, Zinc and Copper Concentration in the Context of Survival in Patients with Laryngeal Cancer

Many factors affect survival, e.g., the stage of the cancer when it was diagnosed, the type of cancer and grade of the cancer cells, the location of the cancer in the larynx, and the general health and fitness of patients. There is no information in the literature about whether and how serum Fe or Cu levels affects the survival rate in humans. It is estimated that for 62-year-old males, 5 year-survival rates for larynx cancer (variants with better prognosis) are 86% for Stage 1, 75% for Stage 2, 59% for Stage 3, and 45% for Stage 4; however, they increase proportionally with time since diagnosis [53]. Among the available literature, we found only one report suggesting that significantly reduced zinc levels in patients with laryngeal cancer may increase patients’ risk of death [54].

Among the patients included in the study with diagnosed laryngeal cancer, 15 of them died (which constituted 37.5% of all analyzed patients). The median survival time from diagnosis was 31.87 ± 21.98 months. On this basis, we decided to analyze the individual parameters of the morphology, immunophenotype and presence of EBV in relation to patients with laryngeal cancer who died and survived more than 60 months during treatment at the Department of Otolaryngology and Oncology, Medical University of Lublin.

The analysis showed that the serum iron and copper levels are statistically significantly lower in patients who died in relation to patients who survived, but also in relation to the control group (Table 13).

Additionally, we conducted an analysis of selected parameters related to EBV infection, which showed a statistically significant increase in the amount of EBV DNA copy number/µg DNA in all the analyzed samples of biological material in patients with laryngeal cancer who died, in patients who survived, and in the control group (Table 14). The correlation analysis performed did not show any significant relationships between serum iron, copper and zinc concentrations and the parameters studied in patients with laryngeal cancer who did not survive. This may be due to the insufficient number of patients. In the case of sick patients who survived more than 60 months, significant negative correlations were observed for EBV in the blood, lymph nodes and tumor tissue (−0.47).

## 4. Discussion

The relationship between Zn, Cu and Fe levels in human body and cancer is complex, and more research is needed to fully understand how these elements affect laryngeal cancer development and progression. These essential nutrients must be obtained from food and drinking water, occasionally as dietary supplements, and are necessary for the body to function normally. High redox potentials of iron and copper, which exist in two oxidation states in biological systems, lead to toxicity in cells and tissues when in excess [55]. High doses of zinc may have harmful effects, including suppressing the immune system and interfering with the absorption of copper and iron [56]. Iron supplements may inhibit intestinal zinc and copper absorption because these elements may compete for binding to a transporter molecule located in the small intestine. Copper-dependent enzymes are needed to transport iron in the body, and a lack of copper causes secondary iron deficiency. Any imbalance in the concentrations of these metals can lead to deficiency and to disease state. On the other hand, the disease itself may be a factor causing deficiencies of various elements, including those described in this study.

There is some scientific evidence that it is possible to distinguish a sample of cancerous tissue from that of a healthy volunteer based on differences in zinc concentrations [57,58,59,60,61]. Reduced levels of this element have been observed in various types of cancers involving, for example, the pancreas, prostate, or breast [62]. Unfortunately, there are still not many available studies describing the relationship between levels of this element and laryngeal cancer. Decreased levels of zinc have been observed in the plasma and whole blood of patients with squamous cell carcinoma of the head and neck, as well as in the hair and nails of patients with cancers involving the head and neck (oral cavity and tongue, salivary glands and larynx) [63,64]. The studies of Dobrowolski et al. comparing the two types of tissues demonstrated that healthy tissues had an increased zinc content of nearly 58% compared to cancerous tissues [65]. In a study conducted by Lubinski J. et al., it has been shown that a deficiency of this metal in the serum of patients is associated with a higher risk of death compared to those with the highest levels tested; in our analyses, we unfortunately did not find confirmation of this [54].

Copper, despite its extremely important role in cellular respiration, free radical detoxification, or cell proliferation, is also suspected of playing a promoter role in cancer formation. Elevated levels of this element in serum or in tumors have been noted in breast, cervical, ovarian, lung, oral, pancreatic, or head and neck cancers [66,67,68,69,70,71]. In our study, the results suggest that the patients were characterized by lower levels of copper compared to healthy volunteers. Among the patients studied, those who survived laryngeal cancer, on the other hand, were characterized by higher levels of this element. The previously mentioned study conducted by Dobrowolski et al. showed that those tissues from healthy parts also showed higher levels of copper [65]. The study conducted by Golasik et al. showed that serum from patients with laryngeal cancer had elevated iron, zinc and copper levels compared to a group of healthy volunteers [72]. The team examined levels of elements not only in serum but also expanded their research to include hair and nail analysis, as blood analysis does not always provide a clear picture of changes occurring throughout the body. Analyses revealed that the hair and nails of healthy subjects showed statistically significantly higher concentrations of elements except zinc. Higher levels of copper and reduced levels of zinc and iron in the serum of laryngeal cancer patients were observed by Rostkowska-Nadolska et al. [73]. In contrast, elevated iron and zinc levels were observed after surgical treatment.

Our findings demonstrate associations between reduced essential element levels and advanced laryngeal cancer. The conducted analyses showed that within the entire research group of individuals with laryngeal cancer, there was a statistically significant positive correlation between the amount of iron, copper and copper/zinc ratio in the serum and the severity of the disease. These elements’ concentration in the serum matrix was statistically significant in the stage of the disease (stage 1 is characterized by slow growth and less susceptibility to spreading, showing higher iron, copper and Cu/Zn concentrations than stage 3, which is characterized by faster growth and a different appearance) (Table 1 and Table 2). There were no statistically significant differences in T (tumor) and N (node) classification (Table 2). In M (metastasis) classification, significant differences were shown: the presence of metastasis in other parts of the body showed a lower content of Fe and Cu in serum than in the absence of spread. Declining iron and copper with higher grades support the possible roles of these elements in cancer progression, potentially through effects on immune function (Table 2). Statistically significant differences were seen between iron and CD4+CD3+ T lymphocytes; CD4/CD8 ratio; CD4+CD69+ tumor; CD4+CD25+ tumor; CD8+PD-1 tumor; CD3+CD25+ node T lymphocytes; and CD19+PD-1 node B lymphocytes (Table 3). Statistically significant differences were seen between zinc and CD19+CD69+ tumor, CD4+CD25+ node T lymphocytes and Cu and: CD3+CD69+, CD4+PD-1, CD8+PD-1 tumor, and CD4+PD-1, CD8+PD-1 node T lymphocytes. There was a statistically significant change in copper/zinc ratio and CD19+CD69+ tumor T lymphocytes (Table 8, Table 9, Table 10 and Table 11). 

The variations observed in the expression of PD-1 receptors across tumors and lymph nodes have significant implications in the realm of cancer immunotherapy. PD-1, a receptor present on cell surfaces, plays a crucial role in regulating T cell exhaustion as a checkpoint. The receptor’s interaction with PD-L1, the programmed death-ligand 1, activates downstream signaling pathways that hinder T cell activation. This mechanism is a key aspect of tumors’ immune evasion strategies [74]. 

The presence of PD-1 in Tregs found in lymph nodes and tumors has been strongly associated with lymph node metastasis. This highlights the significant role PD-1 plays in both the metastatic process and immune suppression within the tumor microenvironment and lymph nodes. As a result, the varying degrees of PD-1 expression in tumor tissue versus lymph nodes may indicate different phases or facets of tumor advancement and interactions with the immune system [75,76,77,78,79].

In the field of cancer treatment, PD-1/PD-L1 checkpoint blockade therapy has become an important cornerstone. It is crucial to comprehend the expression patterns within this context. The aim of these therapies is to alleviate the suppression of tumor cells in the tumor microenvironment. However, it is worth noting that PD-L1 is also found in non-tumor cells like dendritic cells and macrophages. This highlights the intricate interplay in tumor-draining lymph nodes (TDLNs), which are rich in tumor-specific PD-1-positive T cells [80]. The intricate nature of the immunological makeup of tumors and lymph nodes emphasizes the need for tailored therapeutic approaches. With the growing use of PD-1/PD-L1 checkpoint treatment in earlier cancer stages, there is a push to include patients with their tumor-draining lymph nodes intact. This method seeks to fully activate the anti-tumor immune response and maximize the treatment’s clinical advantages [75]. These insights suggest a need for predictive biomarkers and the rational selection of co-treatments to increase the scope and efficacy of immunotherapy targeting the PD-1/PD-L1 checkpoint [81]. 

Regarding cancer, scientists have noticed distinct variations in the presence of the PD-1 molecule in cancerous tissues and lymph nodes. This discovery is essential in creating and utilizing treatments directed at immune checkpoints. Groundbreaking immune checkpoint inhibitors, specifically those that target the PD-1/PD-L1 axis, have transformed cancer therapy by reigniting the body’s natural immune response to fight cancer cells [75,81,82]. 

Our research has revealed that the expression of PD-1 in tumor and lymph node environments exhibits intricate and distinctive patterns. This suggests that the success of checkpoint inhibitors may depend on the location of the tumor and the presence of lymph node metastasis. A personalized approach to immunotherapy is necessary, in which treatment plans are customized based on the patient’s unique tumor immune landscape and lymphatic involvement. With knowledge of the varying PD-1 expression in primary tumors and lymph nodes, medical professionals can more accurately predict which patients will most likely benefit from PD-1 blocking therapies. This is an area of ongoing research for clinicians [75,81].

Furthermore, these discoveries present opportunities for exploring combination therapies. By integrating PD-1 inhibitors with other treatments that specifically address the distinct immune environments of tumors and lymph nodes, we may enhance the efficacy of cancer immunotherapy. Utilizing these combined strategies could potentially overcome resistance mechanisms and enhance clinical outcomes for patients in advanced stages of cancer, including those with lymph node metastasis [75,81].

EBV is important in the pathogenesis and development of laryngeal cancer. Its presence, together with the expression of the PD-1 receptor, influences the development of laryngeal cancer in particular groups of the TNM scale [5]. Elevated PD-1 suggests greater immunosuppression in cancer patients, which could be impacted by low levels of the micronutrients.

In brief, the variance in PD-1 expression between tumors and lymph nodes offers a valuable understanding of tumor–immune interactions, with significant clinical implications. It underscores the need to account for distinct immune microenvironments in various tumor sites when devising and implementing immune checkpoint therapies. These discoveries could facilitate the creation of more efficient and customized treatment approaches for individuals with cancer.

The main limitation of our research was a relatively small sample size. Therefore, the presented research constitutes a pilot study. In the future, we are going to expand the patient group further to increase the accuracy of the values obtained, identify outliers in the data, and ensure smaller measurement error margins. At the same time, we will consider obtaining funds and evaluating patients who have survived a significant period (e.g., five years), which would allow us to understand the progression and changes over time for this type of cancer better.

In the present study, patients’ serum, blood, and lymph nodes were used as research material. As an extension of our research in the future, the tested elements could be determined in biological materials such as other tissue samples. This could provide a more complete understanding of their changes and redistribution in the body. Moreover, the assessment of PD-1/PD-L1 molecule levels currently using flow cytometry and enzyme-linked immunosorbent assays could be enhanced by incorporating molecular techniques to investigate these molecules’ expression levels in immune system cells further.

Completing our future studies with the identified gaps may contribute to a deeper understanding of the complex relationship between trace metal metabolism, the PD-1/PD-L1 pathway, and laryngeal cancer.

We also realize that an inflammatory process complicates the interpretation of iron status measurements. It is unclear which indicators are best suited to assess adequate or excess iron status. Bone marrow iron is the best indicator of the size of the iron store; however, it is not practically measured due to the invasiveness of the procedure itself. Serum Fe is affected by both recent ingestion and inflammation; therefore, it is not a perfect indicator of Fe stores. Our patients were interviewed by a dietician, but a more detailed nutritional assessment may be applied.

In addition, the alteration of iron, copper or zinc levels in patients with laryngeal cancer with respect to controls may be a consequence or a cause of cancer progression, and there may be an association between lower levels and higher-grade tumors and worse prognosis. 

Based on the available evidence on the probable effects of the elements on the immune system and cancer development and the few available reports on the association of PD-1 and its ligand PD-L1 in the development and progression of cancer, especially in the context of nasopharyngeal cancer, it seems important to undertake additional studies to determine the level of PD-L1 expression on selected cells of the immune system and its correlation with the levels of iron, copper and zinc in the body of patients. This would provide important research data on the disruption of the PD-1/PD-L1 pathway and the role of these elements in disrupting anti-tumor immunity [83].

The parameters presented here were determined at the diagnosis of the disease. Future research should also be focused on measuring the fraction of labile iron in plasma, as excess iron may also contribute to oxidative damage. For this reason, further studies on changes in iron metabolism in cancer are needed. We plan to repeat our study in patients who survived the 5-year period. Further studies are needed to clarify the exact role of iron, zinc, and copper metabolism in relation to the immunological and virological parameters in laryngeal cancer patients. The relationships between serum iron, zinc, copper, and the immunopathogenesis of laryngeal cancer merit continued investigation in larger cohorts.

The small number of blood samples needed for testing and their reliable and accurate analysis using the employed analytical technique present the advantages of the study.

## 5. Conclusions

There are noticeable and statistically significant differences between iron, copper and copper/zinc ratio levels for the stage of cancer and within tumor grade and metastases. Surprisingly, parameters related to the presence of EBV, a risk factor for the incidence of laryngeal cancer, were not significantly correlated with serum iron. Our research shows that among patients diagnosed with cancer of the larynx, there are changes in the occurrence of particular subpopulations of T and B lymphocytes compared to the control group. Moreover, these lymphocyte subpopulations of lymph nodes and tumor samples, but not of peripheral blood, correlate significantly with serum iron levels. These changes also concerned the differences in the level of PD-1 receptor expression between the tested patients and the control group, as well as their levels within the tumor and lymph nodes.

Laboratory tests for the assessment of total iron, zinc, and copper content; immune system cells; and tests for EBV infection may prove useful in the future for the diagnosis and management of laryngeal cancer. These tests may allow for quality-of-life improvements in patients through appropriate supplementation and improve the prognosis throughout disease progression. Our findings have demonstrated significant changes that warrant further investigation and confirmation in larger populations. 

## Figures and Tables

**Table 1:** A detailed analysis of selected blood count parameters and serum iron, zinc, copper and Cu/Zn concentration for patients with laryngeal cancer and the control group.

Parameters		Study Group (*n* = 40)		Control Group (*n* = 20)		*p*-Value
		Mean ± SD	Median (Range)	Mean ± SD	Median (Range)	
Selected parameters of peripheral blood counts	WBC [10^3^/mm^3^] Reference range: 4.0–10.0	9.64 ± 2.74	9.65 (2.55–15.78)	7.19 ± 1.64	7.08 (4.12–10.50)	0.0000 ^1^ *
	NEU [10^3^/mm^3^] Reference range: 1.8–7.5	6.89 ± 2.68	6.82 (0.00–13.89)	7.12 ± 2.68	7.05 (0.23–14.12)	0.5539 ^1^
	LYM [10^3^/mm^3^] Reference range: 0.6–3.4	1.92 ± 0.63	1.93 (0.69–3.22)	1.78 ± 0.63	1.79 (0.55–3.08)	0.8699 ^1^
	MON [10^3^/mm^3^] Reference range: 0.0–0.9	0.56 ± 0.19	0.52 (0.00–1.06)	0.60 ± 0.17	0.58 (0.28–0.91)	0.3969 ^1^
	EOS [10^3^/mm^3^] Reference range: 0.0–0.7	0.17 ± 0.13	0.14 (0.00–0.50)	0.21 ± 0.13	0.18 (0.04–0.54)	0.0733 ^2^
	BAS [10^3^/mm^3^] Reference range: 0.0–0.2	0.04 ± 0.02	0.03 (0.01–0.06)	0.03 ± 0.02	0.03 (0.01–0.06)	0.6790 ^2^
	RBC [10^6^/mm^3^] Reference range: 4.5–6.5	4.59 ± 0.52	4.67 (3.09–5.80)	4.80 ± 0.52	4.88 (3.30–6.01)	0.0422 ^2^ *
	HGB [g/dL] Reference range: 13.0–17.5	14.27 ± 1.51	14.35 (9.50–17.70)	15.01 ± 1.19	14.46 (12.36–16.66)	0.0172 ^2^ *
	HCT [%] Reference range: 40–54	42.08 ± 4.74	42.75 (28.00–49.80)	45.90 ± 3.42	45.30 (10.20–56.00)	0.0057 ^2^ *
	MCV [fL] Reference range: 77–93	92.04 ± 5.07	91.53 (80.33–104.53)	94.66 ± 5.02	94.30 (85.50–104.90)	0.9769 ^1^
	MCH [pg] Reference range: 27–32	31.15 ± 1.68	31.68 (26.19–34.37)	31.54 ± 3.78	31.20 (24.39–41.70)	0.5999 ^1^
	MCHC [g/dL] Reference range: 32–38	34.11 ± 3.65	33.25 (30.77–51.01)	32.86 ± 3.63	33.28 (26.36–41.96)	0.9961 ^1^
	RDW [%] Reference range: 10.0–15.0	13.87 ± 1.24	13.60 (11.80–17.70)	12.94 ± 0.96	12.89 (11.39–16.29)	0.0010 ^2^ *
	PLT [10^3^/mm^3^] Reference range: 150–400	260.45 ± 61.73	259.00 (150.00–453.00)	265.45 ± 61.81	265.00 (137.00–440.00)	0.8814 ^1^
Serum Fe (mcg/dL)		79.87 ± 17.28	83.90 (43.93–125.30)	100.86 ± 27.53	96.45 (54.10–186.23)	0.00001 ^2^ *
Serum Zn (mcg/dL)		66.36 ± 14.24	64.40 (43.1–93.3)	83.89 ± 10.16	86.45 (63.8–107.5)	0.00000 ^1^ *
Serum Cu (mcg/dL)		78.24 ± 11.06	75.35 (69.8–125.55)	81.96 ± 12.13	79.22 (61.79–117.2)	0.04760 ^2^ *
Cu/Zn in serum		1.23 ± 0.29	1.18 (0.76–1.84)	0.98 ± 0.13	0.97 (0.71–1.28)	0.00001 ^1^ *

* Statistically significant results. *p* < 0.05. Statistically significant differences were analyzed with the use of ^1^ Student’s *t*-test (d.f. = 58), ^2^ Mann–Whitney U test. Abbreviations: NEU, Neutrophiles; LYM, Lymphocytes; MON, Monocytes; EOS, Eosinophiles; BAS, Basophiles; RBC, Red Blood Cell; HGB, Hemoglobin; HCT, Hematocrit; MCV, Mean Corpuscular Volume; MCH, Mean Corpuscular Hemoglobin; MCHC, Mean Corpuscular Hemoglobin Concentration; RDW, Red Cell Distribution Width; PLT, Platelets.

**Table 2:** Immunophenotyping of peripheral blood, tumor and lymph node lymphocytes.

Frequency of Occurrence [%]		Study Group (*n* = 40)		Control Group (*n* = 20)		*p*-Value
		Mean ± SD	Median (Range)	Mean ± SD	Median (Range)	
Blood Sample	NK cells	14.15 ± 5.58	14.74 (4.16–24.93)	12.69 ± 5.59	13.48 (5.48–23.14)	0.3597 ^1^
	Lymphocytes T CD3+	69.98 ± 7.26	70.66 (57.27–84.20)	76.25 ± 6.66	76.33 (63.34–87.66)	0.0097 ^1^ *
	Lymphocytes B CD19+	14.07 ± 4.18	14.28 (7.11–22.66	9.46 ± 4.38	8.47 (4.35–22.12)	0.0005 ^1^ *
	Lymphocytes T CD4+ CD3+	43.55 ± 6.61	42.89 (29.91–63.96)	48.96 ± 5.89	47.04 (38.97–61.22)	0.0031 ^2^ *
	Lymphocytes T CD8+ CD3+	26.46 ± 6.32	25.44 (16.66–41.76)	27.01 ± 4.51	26.86 (21.31–36.71)	0.4681 ^1^
	Ratio of lymphocytes T CD4+ CD3+ to CD8+ CD3+	1.77 ± 0.61	1.69 (0.72–3.84)	1.87 ± 0.39	1.80 (1.13–2.69)	0.2607 ^2^
	Lymphocytes T CD3+ CD69+	5.50 ± 3.08	4.87 (1.40–12.32)	3.07 ± 1.21	2.38 (1.77–5.74)	0.0061 ^2^ *
	Lymphocytes B CD19+ CD69+	6.07 ± 3.19	5.42 (1.32–15.42)	6.74 ± 2.71	6.53 (2.17–15.40)	0.3554 ^2^
	Lymphocytes T CD3+ CD25+	45.05 ± 13.86	45.44 (12.97–89.93)	31.34 ± 6.01	30.13 (23.46–50.20)	0.001 ^2^ *
	Lymphocytes B CD19+ CD25+	31.20 ± 11.57	28.53 (10–74.64	30.22 ± 10.71	29.10 (12.04–45.48)	0.9205 ^2^
	Lymphocytes T CD4+ CD69+	5.34 ± 2.85	4.70 (1.23–13.84)	3.27 ± 1.99	2.19 (1.17–7.58)	0.0058 ^2^ *
	Lymphocytes T CD8+ CD69+	4.49 ± 2.36	3.69 (1.06–10.99	1.41 ± 1.05	1.19 (0.29–4.19)	0.0000 ^2^ *
	Lymphocytes T CD4+ CD25+	56.32 ± 16.01	53.32 (29.61–96.69)	46.90 ± 8.41	45.60 (29.98–67.74)	0.0262 ^2^ *
	Lymphocytes T CD8+ CD25+	8.77 ± 8.46	5.97 (1.45–41.06)	1.97 ± 0.88	1.91 (0.55–4.22)	0.0000 ^2^ *
	Lymphocytes B CD19+ PD-1+	4.29 ± 1.90	4.20 (1.14–10.87)	6.42 ± 3.19	7.11 (1.73–11.34)	0.0330 ^2^ *
	Lymphocytes T CD4+ PD-1+	21.39 ± 5.28	21.44 (12.21–32.16)	7.57 ± 1.63	7.49 (5.09–10.24)	0.0000 ^1^ *
	Lymphocytes T CD8+ PD-1+	18.15 ± 5.83	17.19 (10.12–32.45)	4.33 ± 2.08	4.02 (1.32–8.36)	0.0000 ^2^ *
Tumor Sample	Lymphocytes T CD3+ CD69+	40.08 ± 14.83	39.59 (5.63–74.51)	N/A	N/A	N/A
	Lymphocytes B CD19+ CD69+	26.76 ± 12.69	24.01 (10.87–56.67)	N/A	N/A	N/A
	Lymphocytes T CD3+ CD25+	30.92 ± 15.04	28.87 (9.25–67.18)	N/A	N/A	N/A
	Lymphocytes B CD19+ CD25+	27.75 ± 20.56	19.95 (4.67–81.11)	N/A	N/A	N/A
	Lymphocytes T CD4+ CD69+	46.24 ± 18.01	45.50 (15.67 –81.51)	N/A	N/A	N/A
	Lymphocytes T CD8+ CD69+	42.59 ± 21.54	39.07 (11.25–89.92)	N/A	N/A	N/A
	Lymphocytes T CD4+ CD25+	28.87 ± 15.81	26.82 (6.57–64.14)	N/A	N/A	N/A
	Lymphocytes T CD8+ CD25+	9.55 ± 4.82	8.34 (2.12–21.12)	N/A	N/A	N/A
	Lymphocytes B CD19+ PD-1	26.41 ± 9.90	22.86 (12.25–50.25)	N/A	N/A	N/A
	Lymphocytes T CD4+ PD-1+	41.64 ± 16.88	36.97 (15.89–75.45)	N/A	N/A	N/A
	Lymphocytes T CD8+ PD-1+	42.82 ± 17.73	38.02 (10.45–79.45)	N/A	N/A	N/A
Lymph Node Sample	Lymphocytes T CD3+ CD69+	26.31 ± 16.03	25.39 (2.74–76.58)	N/A	N/A	N/A
	Lymphocytes B CD19+ CD69+	25.25 ± 11.07	23.61 (10.23–65.15)	N/A	N/A	N/A
	Lymphocytes T CD3+ CD25+	28.74 ± 10.04	28.88 (7.36–51.72)	N/A	N/A	N/A
	Lymphocytes B CD19+ CD25+	24.12 ± 8.35	22.20 (12.21–39.92)	N/A	N/A	N/A
	Lymphocytes T CD4+ CD69+	16.21 ± 9.89	13.57 (2.85–38.59)	N/A	N/A	N/A
	Lymphocytes T CD8+ CD69+	15.93 ± 10.38	15.43 (2.39–47.97)	N/A	N/A	N/A
	Lymphocytes T CD4+ CD25+	33.97 ± 11.42	31.91 (10.23–60.47)	N/A	N/A	N/A
	Lymphocytes T CD8+ CD25+	7.43 ± 5.74	6.06 (1.96–34.16)	N/A	N/A	N/A
	Lymphocytes B CD19+ PD-1	10.37 ± 5.49	8.71 (3.47–22.14)	N/A	N/A	N/A
	Lymphocytes T CD4+ PD-1+	21.63 ± 9.46	18.70 (9.18–42.87)	N/A	N/A	N/A
	Lymphocytes T CD8+ PD-1+	19.43 ± 6.94	20.07 (7.12–32.46)	N/A	N/A	N/A

* Statistically significant results, *p* < 0.05; p N/A- not applicable. Statistically significant differences were analyzed with the use of ^1^ Student’s *t*-test (d.f. = 58), ^2^ Mann–Whitney U test.

**Table 3:** Relationships among serum Fe, Zn, Cu and Cu/Zn and grade/stage of the disease and TNM scale.

**Grade**	**G1**		**G2**			**G3**		**ANOVA** ***p*-Value**	**G1 vs. G2**	**G1 vs. G3**	**G2 vs. G3**
**Parameters**	**Men ± SD**	**Median (Range)**	**Mean ± SD**	**Median (Range)**		**Mean ± SD**	**Median (Range)**				
Serum Fe	90.48 ± 13.51	89.4 (73.8–125.3)	76.34 ± 19.26	83.9 (43.93–102)		74.17 ± 15.25	78.1 (45.87–91.6)	0.0364 ^1^ *	0.0807	0.046 *	0.9356
Serum Zn	62.12 ± 11.2	60.4 (45.3–85.56)	67.61 ± 13.4	68 (43.2–90.7)		68.82 ± 17.56	64.59 (43.1–93.3)	0.4677 ^1^	NS	NS	NS
Serum Cu	85.33 ± 15.59	79.3 (74.2–125.55)	77 ± 8.05	76.4 (69.9–102.2)		73.12 ± 4.29	70.7 (69.8–83.9)	0.0064 ^2^ *	0.2317	0.0045 *	0.3625
Cu/Zn	1.4 ± 0.27	1.4 (0.95–1.84)	1.17 ± 0.23	1.15 (0.79–1.67)		1.13 ± 0.31	1.13 (0.75–1.84)	0.027 ^2^ *	0.0879	0.0374 *	1.0
Statistically significant results, *p* < 0.05; Statistically significant differences were analyzed with the use of ^1^ ANOVA test, ^2^ ANOVA Kruskal–Wallis test with post hoc tests to confirm where the differences occurred.											
**Tumor—the Size of the Primary Tumor**		**T2**				**T3**		**T4**			**ANOVA *p*-Value**
**Parameters**		**Mean ± SD**		**Median (Range)**		**Mean ± SD**	**Median (Range)**	**Mean ± SD**	**Median (Range)**		
Serum Fe		76.41 ± 25.25		81.67 (45.7–102)		77.14 ± 16.31	82.5 (43.93–96)	82.56 ± 16.2	84.9 (45.87–125.3)		0.7862 ^2^
Serum Zn		69.1 ± 8.44		67.81 (60.4–79.45)		67.14 ± 18	62.3 (43.1–90.7)	65.09 ± 13.38	64.2 (43.2–93.3)		0.8152 ^1^
Serum Cu		80.17 ± 10.26		78.8 (70.4–98.9)		82.04 ± 15.34	76.45 (70.5–125.55)	75.33 ± 7.19	73.7 (69.8–101.6)		0.1264 ^2^
Cu/Zn		1.17 ± 0.2		1.11 (0.98–1.49)		1.29 ± 0.35	1.23 (0.78–1.84)	1.2 ± 0.26	1.16 (0.75–1.84)		0.6009 ^1^
Statistically significant results, *p* < 0.05; Statistically significant differences were analyzed with the use of ^1^ ANOVA test, ^2^ ANOVA Kruskal–Wallis test with post hoc tests to confirm where the differences occurred.											
**Nodules—Metastases in the Lymph Nodes**		**N0**		**N1**		**N2**		**N3**			**ANOVA *p*-Value**
**Parameters**		**Mean ± SD**	**Median (Range)**	**Mean ± SD**	**Median (Range)**	**Mean ± SD**	**Median (Range)**	**Mean ± SD**	**Median (Range)**		
Serum Fe		78.83 ± 21.24	85.25 (43.93–102)	84.17 ± 17.27	83.7 (54.78–125.3)	79.52 ± 15.2	84.3 (45.87–94.6)	70.51 ± 22.07	69.67 (45.7–97)		0.8511 ^2^
Serum Zn		61.94 ± 14.39	60.43 (45.3–90.7)	65.61 ± 10.55	62.05 (52–85.56)	65.15 ± 15.47	64.4 (43.1–92.45)	82.27 ± 12.27	84.63 (66.51–93.3)		0.1119 ^1^
Serum Cu		78.69 ± 9.99	75.9 (70.4–102.2)	79.79 ± 14.98	75.88 (69.9–125.55)	76.6 ± 8.08	74.05 (69.8–101.6)	79.23 ± 13.49	73.8 (70.4–98.9)		0.9067 ^2^
Cu/Zn		1.31 ± 0.24	1.26 (1.02–1.67)	1.23 ± 0.21	1.22 (0.95–1.74)	1.24 ± 0.34	1.15 (0.76–1.84)	1 ± 0.34	0.88 (0.75–1.49)		0.3673 ^1^
* Statistically significant results, *p* < 0.05; Statistically significant differences were analyzed with the use of ^1^ ANOVA test, ^2^ ANOVA Kruskal–Wallis test with post hoc tests to confirm where the differences occurred.											
**Metastases—Distant (Organ) Metastases**		**M0**				**M1**					***p*** **-Value**
**Parameters**		**Mean ± SD**		**Median (Range)**		**Mean ± SD**		**Median (Range)**			
Serum Fe		84.81 ± 14.6		85.45 (45.7–125.3)		65.05 ± 17.74		58.52 (43.93–91.6)			0.0076 ^2^ *
Serum Zn		65.73 ± 13.15		64.1 (43.1–90.7)		68.25 ± 17.8		64.4 (46.3–93.3)			0.6345 ^1^
Serum Cu		80.15 ± 12.11		76.75 (69.9–125.55)		72.5 ± 2.88		70.8 (69.8–77.3)			0.0125 ^2^ *
Cu/Zn		1.26 ± 0.28		1.2 (0.79–1.84)		1.13 ± 0.3		1.13 (0.75–1.67)			0.2235 ^1^
* Statistically significant results, *p* < 0.05; Statistically significant differences were analyzed with the use of ^1^ Student’s *t*-test (d.f. = 38). ^2^ Mann–Whitney U test.											

**Table 4:** Basic parameters related to the presence of EBV in the tested biological material in patients diagnosed with laryngeal cancer and the control group.

Parameters	Study Group (*n* = 40)		Control Group (*n* = 20)		*p*-Value
	Mean ± SD	Median (Range)	Mean ± SD	Median (Range)	
EBV DNA copy number/µg DNA in the tumor tissue	266.82 ± 361.23	37.88 (0.00–1212.28)	0.00 ± 0.00	0.00 (0.00–0.00)	0.0000 *
EBV DNA copy number/µg DNA in the lymph node	203.69 ± 286.92	29.95 (0.00–863.08)	0.00 ± 0.00	0.00 (0.00–0.00)	0.0000 *
EBV DNA copy number/µg DNA in the blood	117.96 ± 176.25	16.24 (0.00–675.30)	0.00 ± 0.00	0.00 (0.00–0.00)	0.0000 *
IgM VCA EBV	24.19 ± 8.98	21.49 (15.31–49.74)	0.00 ± 0.00	0.00 (0.00–0.00)	0.0000 *
IgG VCA EBV	91.52 ± 51.17	77.32 (26.22–242.62)	56.39 ± 22.19	53.87 (25.58–89.74)	0.0100 *

* Statistically significant results, *p* < 0.05; Statistically significant differences were analyzed with the use of Mann–Whitney U test.

**Table 5:** Comparative analysis of tested immune system parameters in patients with laryngeal cancer by EBV activity or absence.

Lymphocyte subpopulations	**Parameters**	**Study Group**				**Control Group**		**ANOVA**	***p*-Value**		
		**EBV+** **(*n* = 20)**		**EBV−** **(*n* = 20)**		**EBV−** **(*n* = 20)**		***p*-Value**	**Study Group EBV+ vs. EBV−**	**Study Group EBV+ vs. Control Group EBV−**	**Study Group EBV− vs. Control Group EBV−**
		**Mean ± SD**	**Median (Range)**	**Mean ± SD**	**Median (Range)**	**Mean ± SD**	**Median (Range)**				
	T CD3+	69.55 ± 7.11	70.89 (59.02–81.68)	70.41 ± 7.74	70,44 (57.27–84.20)	76.10 ± 6.46	76.11 (63.34–87.66)	0.00995 ^1^ *	0.71523	0.00415 *	0.01598 *
	B CD19+	13.34 ± 4.18	13.46 (7.11–22.15)	14.81 ± 4.26	14.48 (8.11–22.66)	10.26 ± 4.54	9.94 (4.35–22.12)	0.00529 ^1^ *	0.27644	0.03177 *	0.00229 *
	T CD4+CD3+	42.26 ± 5.89	41.94 (29.91–57.94)	44.83 ± 7.34	45.40 (33.17–63.96)	49.29 ± 5.81	48.29 (38.97–61.22)	0.00174 ^2^ *	0.20358	0.00056 *	0.02072 *
	T CD3+CD69+ blood	4.46 ± 2.68	3.39 (1.40–10.85)	6.54 ± 3.25	6.64 (1.72–12.32)	3.08 ± 1.16	2.61 (1.77–5.74)	0.00296 ^2^ *	0.06787	0.04375 *	0.00016 *
	B CD19+CD69+ blood	5.04 ± 2.40	5.17 (1.32–9.12)	7.10 ± 3.66	6.23 (1.77–15.42)	6.82 ± 2.75	6.76 (2.17–15.40)	0.04961 ^2^ *	0.04323 *	0.04987 *	0.88172
	T CD3+CD25+ blood	42.26 ± 11.34	45.44 (12.97–56.16)	47.85 ± 16.11	45.39 (26.15–89.93)	31.85 ± 7.25	30.02 (23.46–51.12)	0.00041 ^2^ *	0.21346	0.00112 *	0.00051 *
	CD4+CD69+ blood	5.27 ± 2.99	4.44 (2.00–13.84)	5.41 ± 2.86	4.84 (1.23–10.11)	3.08 ± 1.90	2.14 (1.17–7.58)	0.00392 ^2^ *	0.87964	0.00333 *	0.00579 *
	CD8+CD69+ blood	4.71 ± 2.38	4.35 (1.06–8.85)	4.28 ± 2.45	3.66 (1.09–10.99)	1.29 ± 1.00	1.10 (0.29–4.19)	0.00001 ^2^ *	0.57918	0.00000 *	0.00002 *
	CD4+CD25+ blood	53.48 ± 14.22	49.49 (29.61–76.88)	59.17 ± 17.91	56.58 (31.14–96.69)	46.82 ± 8.60	45.10 (29.98–67.74)	0.03072 ^2^ *	0.27337	0.07643	0.01436 *
	CD8+CD25+ blood	9.50 ± 9.28	7.25 (1.45–41.06)	8.04 ± 7.97	5.88 (1.76–35.94)	2.02 ± 1.03	1.76 (0.55–4.59)	0.00003 ^2^ *	0.57920	0.00000 *	0.00001 *
	B CD19+PD-1	4.11 ± 1.70	3.78 (1.89–7.18)	4.48 ± 2.15	4.20 (1.14–10.87)	6.58 ± 3.08	7.11 (1.73–11.34)	0.02350 ^2^ *	0.66512	0.00979 *	0.04112 *
	CD4+PD-1 blood	23.26 ± 4.47	22.13 (15.67–32.16)	19.53 ± 5.60	17.93 (12.21–30.27)	7.74 ± 1.66	7.80 (5.09–10.24)	0.00000 ^1^ *	0.02530*	0.00000 *	0.00000 *
	CD8+PD-1 blood	19.54 ± 7.36	17.45 (10.12–32.45)	16.77 ± 3.65	16.50 (11.45–25.26)	4.22 ± 2.07	4.02 (1.32–8.36)	0.00000 ^2^ *	0.42482	0.00000 *	0.00000 *
	IgG VCA EBV quantitative	83.04 ± 35.91	77.04 (31.56–148.30)	99.99 ± 63.81	77.77 (26.22–242.62)	58.71 ± 21.44	60.33 (20.58–89.74)	0.04617 ^2^ *	0.30895	0.03851 *	0.05652

* Statistically significant results, *p* < 0.05; Statistically significant differences were analyzed with the use of ^1^ ANOVA test, ^2^ ANOVA Kruskal–Wallis test with post hoc tests to confirm where the differences occurred.

**Table 6:** Comparative analysis of tested elements levels in patients with laryngeal cancer by EBV activity or absence.

Parameters	Study Group				Control Group		ANOVA	Post-hoc *p*-Value		
	EBV+ (*n* = 20)		EBV− (*n* = 20)		EBV−		*p*-Value	Study Group EBV+ vs. EBV−	Study Group EBV+ vs. Control Group EBV−	Study Group EBV− vs. Control Group EBV−
	Mean ± SD	Median (Range)	Mean ± SD	Median (Range)	Mean ± SD	Median (Range)				
Serum Fe (mcg/dL)	69.16 ± 16.81	73.75 (43.93–96.40)	90.59 ± 10.23	89.50 (75.30–125.30)	100.86 ± 27.86	96.45 (54.10–186.23)	0.00001 ^2^ *	0.00007 *	0.00002 *	0.16045
Serum Zn (mcg/dL)	66.50 ± 13.75	63.25 (46.30–93.30)	66.22 ± 15.07	66.46 (43.10–90.70)	83.89 ± 10.16	86.45 (63.80–107.50)	0.00101 ^1^ *	0.95238	0.00002 *	0.00006 *
Serum Cu (mcg/dL)	75.71 ± 7.28	73.95 (69.80–101.60)	80.76 ± 13.59	75.90 (69.90–125.55)	81.96 ± 12.13	79.22 (61.79–117.20)	0.04998 ^2^ *	0.15167	0.01826 *	0.39271
Cu/Zn in serum	1.19 ± 0.28	1.18 (0.76–1.84)	1.27 ± 0.30	1.18 (0.79–1.84)	0.98 ± 0.13	0.97 (0.71–1.28)	0.00577 ^1^ *	0.36958	0.00481 *	0.00043 *

* Statistically significant results. *p* < 0.05; Statistically significant differences were analyzed with the use of ^1^ ANOVA test, ^2^ ANOVA Kruskal–Wallis test with post hoc tests to confirm where the differences occurred.

**Table 7:** Correlations between total content of tested elements in serum and other parameters of the immune system.

Characteristics of the Basic Parameters of the Immune System								
		Age	NK	Lymphocytes T CD3+	Lymphocytes B CD19+	Lymphocytes T CD4+CD3+	Lymphocytes T CD8+CD3+	Ratio CD4/CD8
Serum Fe	r	−0.1600	−0.2334	0.1832	−0.0108	0.3674	−0.1699	0.3847
	*p*	0.324	0.147	0.258	0.947	0.020 *	0.295	0.014 *
Serum Zn	*r*	0.236	0.036	0.065	−0.048	−0.009	0.133	−0.109
	*p*	0.14227	0.82444	0.69022	0.76893	0.95624	0.41352	0.50312
Serum Cu	*r*	−0.036	-0.176	0.121	0.039	0.195	0.041	0.052
	*p*	0.82522	0.27721	0.45754	0.81320	0.22674	0.79985	0.74786
Cu/ Zn in serum	*r*	−0.151	−0.112	−0.046	0.132	0.082	−0.168	0.178
	*p*	0.35169	0.49066	0.77998	0.41750	0.61661	0.29979	0.27220

* Statistically significant results. *p* < 0.05; Spearman correlation coefficient and *t*-test for correlation were used to determine if a correlation coefficient is statistically significant.

**Table 8:** Correlations between serum iron and other parameters of the immune system in blood. tumor and node samples.

Lymphocyte Subpopulations																						
	CD3+CD69+		CD19+CD69+		CD3+CD25+		CD19+CD25+		CD4+CD69+		CD8+CD69+		CD4+CD25+		CD8+CD25+		CD19+PD-1+		CD4+PD-1+		CD8+PD-1+	
Serum Fe	r	*p*	r	*p*	r	*p*	r	*p*	r	*p*	r	*p*	r	*p*	r	*p*	r	*p*	r	*p*	r	*p*
Blood samples	0.257	0.109	0.165	0.310	0.101	0.534	−0.171	0.290	0.187	0.248	0.092	0.571	0.064	0.697	−0.187	0.249	0.213	0.186	−0.180	0.267	−0.042	0.799
Tumor samples	0.316	0.04708 *	−0.056	0.733	0.273	0.088	0.143	0.378	0.386	0.014 *	0.210	0.194	0.360	0.023 *	0.105	0.520	0.039	0.814	−0.263	0.101	−0.359	0.023 *
Node samples	−0.042	0.795	0.225	0.163	0.371	0.018 *	−0.047	0.772	−0.109	0.503	−0.005	0.978	0.306	0.055	−0.210	0.193	0.127	0.434	−0.529	0.000 *	−0.036	0.827

* Statistically significant results. *p* < 0.05; Spearman correlation coefficient and *t*-test for correlation were used to determine if a correlation coefficient is statistically significant.

**Table 9:** Correlations between serum zinc and other parameters of the immune system in blood. tumor and node samples.

Lymphocyte Subpopulations																						
	CD3+CD69+		CD19+CD69+		CD3+CD25+		CD19+CD25+		CD4+CD69+		CD8+CD69+		CD4+CD25+		CD8+CD25+		CD19+PD-1+		CD4+PD-1+		CD8+PD-1+	
Serum Zn	r	*p*	r	*p*	r	*p*	r	*p*	r	*p*	r	*p*	r	*p*	r	*p*	r	*p*	r	*p*	r	*p*
Blood samples	−0.020	0.90396	−0.056	0.73095	0.020	0.90305	0.027	0.86946	0.060	0.71420	−0.059	0.71756	−0.061	0.70771	0.022	0.89486	−0.054	0.73875	0.110	0.50120	−0.027	0.86809
Tumor samples	−0.147	0.36441	−0.340	0.03190 *	−0.003	0.98579	0.058	0.72275	−0.032	0.84464	0.006	0.97296	0.121	0.45789	0.144	0.37382	−0.062	0.70358	0.131	0.42154	−0.100	0.53803
Node samples	−0.042	0.79634	−0.103	0.52683	−0.244	0.12914	0.029	0.85836	0.067	0.68301	−0.098	0.54880	0.327	0.03918 *	−0.079	0.62679	0.074	0.64983	−0.173	0.28602	−0.042	0.79833

* Statistically significant results. *p* < 0.05; Spearman correlation coefficient and *t*-test for correlation were used to determine if a correlation coefficient is statistically significant.

**Table 10:** Correlations between serum copper and other parameters of the immune system in blood. tumor and node samples.

Lymphocyte Subpopulations																						
	CD3+CD69+		CD19+CD69+		CD3+CD25+		CD19+CD25+		CD4+CD69+		CD8+CD69+		CD4+CD25+		CD8+CD25+		CD19+PD-1+		CD4+PD-1+		CD8+PD-1+	
Serum Cu	r	*p*	r	*p*	r	*p*	r	*p*	r	*p*	r	*p*	r	*p*	r	*p*	r	*p*	r	*p*	r	*p*
Blood samples	−0.088	0.58834	0.026	0.87123	0.031	0.84910	−0.263	0.10102	−0.280	0.08061	−0.218	0.17695	0.125	0.44254	−0.233	0.14765	−0.215	0.18263	−0.160	0.32366	−0.233	0.14744
Tumor samples	0.364	0.02090 *	0.203	0.20979	0.104	0.52164	0.137	0.39839	0.306	0.05492	0.103	0.52808	0.134	0.41076	0.302	0.05832	−0.204	0.20649	−0.491	0.00131 *	−0.339	0.03224 *
Node samples	−0.061	0.70890	−0.143	0.37721	0.182	0.26184	−0.014	0.93357	0.039	0.81277	−0.047	0.77129	0.026	0.87531	0.272	0.08980	−0.076	0.64021	−0.399	0.01074 *	−0.459	0.00288 *

* Statistically significant results. *p* < 0.05; Spearman correlation coefficient and *t*-test for correlation were used to determine if a correlation coefficient is statistically significant.

**Table 11:** Correlations between copper/zinc ratio and other parameters of the immune system in blood. tumor and node samples.

Lymphocyte Subpopulations																						
	CD3+CD69+		CD19+CD69+		CD3+CD25+		CD19+CD25+		CD4+CD69+		CD8+CD69+		CD4+CD25+		CD8+CD25+		CD19+PD-1+		CD4+PD-1+		CD8+PD-1+	
Cu/Zn in serum	r	*p*	r	*p*	r	*p*	r	*p*	r	*p*	r	*p*	r	*p*	r	*p*	r	*p*	r	*p*	r	*p*
Blood samples	−0.008	0.96243	0.064	0.69488	0.068	0.67457	−0.075	0.64450	−0.139	0.39066	−0.045	0.78173	0.223	0.16684	−0.074	0.64865	−0.010	0.95006	−0.090	0.57965	−0.077	0.63745
Tumor samples	0.269	0.09331	0.395	0.01164 *	0.055	0.73792	−0.071	0.66281	0.126	0.43888	0.024	0.88488	−0.044	0.78699	0.024	0.88213	−0.025	0.87669	−0.310	0.05147	−0.039	0.80968
Node samples	0.066	0.68725	0.105	0.51800	0.226	0.16101	−0.067	0.68169	0.057	0.72580	0.042	0.79768	−0.250	0.12047	0.033	0.83880	−0.096	0.55730	−0.006	0.96975	−0.122	0.45413

* Statistically significant results. *p* < 0.05; Spearman correlation coefficient and *t*-test for correlation were used to determine if a correlation coefficient is statistically significant.

**Table 12:** Correlations between tested elements levels and parameters related to the presence of EBV.

Characteristics of Parameters Related to the Presence of EBV						
		EBV DNA Copy Number/µg DNA in the Tumor Tissue	EBV DNA Copy Number/µg DNA in the Lymph Node	EBV DNA Copy Number/µg DNA in the Blood	IgM VCA EBV Quantitatively	IgG VCA EBV Quantitatively
Serum Fe	r	0.3319	0.3518	0.1723	0.2457	0.1085
	*p*	0.153	0.128	0.468	0.296	0.505
Serum Zn	*r*	−0.105	−0.096	−0.140	−0.183	−0.026
	*p*	0.658	0.68649	0.55649	0.43879	0.91480
Serum Cu	*r*	−0.243	−0.236	−0.234	−0.522	0.150
	*p*	0.30183	0.31592	0.32070	0.01818 *	0.52654
Cu/ Zn in serum	*r*	0.054	0.048	0.078	0.048	−0.035
	*p*	0.82067	0.84034	0.74315	0.84034	0.88490

* Statistically significant results. *p* < 0.05; Spearman correlation coefficient and *t*-test for correlation were used to determine if a correlation coefficient is statistically significant.

**Table 13:** Analysis of selected parameters of peripheral blood count and serum iron concentration in patients with laryngeal cancer accounting for the survival status in relation to control patients.

Parameters	Study Group (*n* = 40)				Nonsurvivng vs. Surviving Patients *p*-Value
	Nonsurivivng Patients (*n* = 10)		Surviving Patients (*n* = 30)		
	Mean ± SD	Median (Range)	Mean ± SD	Median (Range)	
WBC [10^3^/mm^3^]	9.52 ± 3.26	9.18 (5.83–15.78)	9.67 ± 2.66	9.69 (2.55–15.78)	0.89510 ^1^
NEU [10^3^/mm^3^]	6.78 ± 3.45	5.81 (3.16–13.89)	6.93 ± 2.49	6.91 (0–13.89)	0.88470 ^1^
LYM [10^3^/mm^3^]	1.83 ± 0.52	1.84 (1.12–3.08)	1.76 ± 0.68	1.63 (0.55–2.98)	0.78365 ^1^
MON [10^3^/mm^3^]	0.59 ± 0.20	0.52 (0.42–1.06)	0.55 ± 0.19	0.52 (0–0.89)	0.55694 ^1^
EOS [10^3^/mm^3^]	0.18 ± 0.15	0.14 (0.03–0.50)	0.16 ± 0.13	0.14 (0–0.5)	0.88804 ^2^
BAS [10^3^/mm^3^]	0.03 ± 0.01	0.03 (0.01–0.06)	0.04 ± 0.02	0.04 (0.01–0.07)	0.31570 ^2^
NEU [%]	67.73 ± 12.68	67.45 (53.50–88.00)	69.18 ± 15.73	73.70 (0–90.60)	0.44961 ^2^
LYM [%]	21.83 ± 9.49	22.15 (7.10–32.50)	21.16 ± 16.10	17.80 (4.90–98.40)	0.44963 ^2^
MON [%]	6.51 ± 2.11	6.00 (4.00–10.90)	5.79 ± 2.01	5.50 (0–10.40)	0.44947 ^2^
EOS [%]	1.91 ± 1.48	1.55 (0.2–4.00)	1.75 ± 1.54	1.30 (0–6.80)	0.77186 ^2^
BAS [%]	0.35 ± 0.15	0.30 (0.10–0.60)	0.45 ± 0.31	0.40 (0.1–1.6)	0.49357 ^2^
RBC [10^6^/mm^3^]	4.70 ± 0.26	4.71 (4.36–5.06)	4.56 ± 0.59	4.59 (3.09–5.8)	0.46256 ^2^
HGB [g/dl]	14.12 ± 0.81	14.40 (12.60–15.00)	14.32 ± 1.72	14.25 (9.5–17.7)	0.87575 ^2^
HCT [%]	42.98 ± 2.49	44.15 (38.70–45.30)	43.37 ± 5.25	44.2 (28–54.7)	0.67312 ^2^
MCV [fL]	92.91 ± 4.42	93.70 (87.30–101.60)	95.25 ± 5.22	95.4 (85.5–104.9)	0.18393 ^1^
MCH [Pg]	30.50 ± 1.69	30.55 (28.30–32.60)	31.51 ± 1.39	31.8 (28.4–34.2)	0.10945 ^1^
MCHC [g/dL]	32.80 ± 0.74	32.55 (32.00–34.20)	33.15 ± 1.00	33.2 (30.9–35.5)	0.25492 ^1^
RDW [%]	13.80 ± 1.14	13.55 (12.90–16.60)	13.80 ± 1.35	13.55 (11.8–17.7)	0.93766 ^2^
PLT [10^3^/mm^3^]	255.60 ± 50.59	260.00 (150.00–334.00)	262.07 ± 66.72	259 (150–453)	0.91289 ^1^
MPV [fL]	7.39 ± 1.20	6.85 (6.30–9.40)	7.67 ± 1.12	7.45 (6.2–10.6)	0.38111 ^2^
Creatinine [mg/dl]	0.65 ± 0.15	0.60 (0.5–1.00)	0.73 ± 0.23	0.7 (0.5–1.6)	0.25299 ^2^
Serum Fe [mcg/dL]	66.89 ± 16.05	66.93 (43.93–91.60)	87.66 ± 13.38	87.4 (45.7–125.3)	0.00039 ^2^ *
Serum Zn [mcg/dL]	66.44 ± 16.02	64.2 (45.3–93.3)	66.31 ± 13.42	66.4 (43.1–90.7)	0.97725 ^1^
Serum Cu [mcg/dL]	72.88 ± 3.66	70.7 (69.8–82.1)	81.45 ± 12.74	78.1 (70.5–125.55)	0.00119^2^ *
Cu/Zn in serum	1.16 ± 0.29	1.13 (0.76–1.67)	1.27 ± 0.28	1.21 (0.79–1.84)	0.23425 ^1^

* Statistically significant results. *p* < 0.05; Statistically significant differences were analyzed with the use of ^1^ Student’s *t*-test (d.f. = 38). ^2^ Mann–Whitney U test. Abbreviations: NEU. Neutrophiles; LYM. Lymphocytes; MON. Monocytes; EOS. Eosinophiles; BAS. Basophiles; RBC. Red Blood Cell; HGB. Hemoglobin; HCT. Hematocrit; MCV. Mean Corpuscular Volume; MCH. Mean Corpuscular Hemoglobin; MCHC. Mean Corpuscular Hemoglobin Concentration; RDW. Red Cell Distribution Width; PLT. Platelets.

**Table 14:** Analysis of selected parameters related to EBV infection in patients with laryngeal cancer. including the survival status and the control group.

Parameters	Study Group (*n* = 40)				Nonsurviving vs. Surviving Patients *p*-Value
	Nonsurviving Patients (*n* = 15)		Surviving Patients (*n* = 25)		
	Mean ± SD	Median (Range)	Mean ± SD	Median (Range)	
EBV DNA copy numer/µg DNA in the tumor tissue	509.80 ± 433.43	367.46 (0–1212.28)	121.04 ± 221.03	0 (0–635.66)	0.00043 *
EBV DNA copy numer/ µg DNA in the lymph node	401.11 ± 355.42	232.52 (0–863.08)	85.21 ± 156.64	0 (0–456.35)	0.00038 *
EBV DNA copy numer/µg DNA in the blood	248.20 ± 223.95	126.59 (0–675.30)	39.82 ± 74.53	0 (0–272.82)	0.00027 *
IgM VCA EBV	26.12 ± 10.10	23.3 (15.31–49.74)	20.61 ± 6.46	17.59 (15.4–30)	0.23458
IgG VCA EBV	76.82 ± 39.24	72.65 (31.56–148.3)	100.33 ± 57.01	90.08 (26.22–242.62)	0.15422

* Statistically significant results. *p* < 0.05; Statistically significant differences were analyzed with the use of Mann–Whitney U test.

## Data Availability

The data presented in this study are available on request from the second and the last authors.

## Supplementary Materials

The following supporting information can be downloaded at: https://www.mdpi.com/article/10.3390/jcm13020511/s1, Table S1: Comparative analysis of tested immune system parameters in patients with laryngeal cancer by EBV activity or absence.
