# Supplementary material for: The Relationship between Cancer Stage, Selected Immunological Parameters, Epstein–Barr Virus Infection, and Total Serum Content of Iron, Zinc, and Copper in Patients with Laryngeal Cancer
Source: J Clin Med. 2024 Jan 16;13(2):511. doi: 10.3390/jcm13020511 (PMC10816330; doi:10.3390/jcm13020511)
Supplement: Supplementary file 1 [file jcm-13-00511-s001.zip › jcm-2786494-supplementary.pdf]

Table S1. Comparative analysis of tested immune system parameters in patients with laryngeal cancer by EBV activity or absence.

	Parameters	Study group				Control group		ANOVA	p- value		
		EBV+ (n=20)		EBV- (n=20)		EBV-		p- value	Study group EBV+ vs. EBV-	Study group EBV+ vs. control group EBV-	Study group EBV- vs. control group EBV-
		Mean±SD	Median (Range)	Mean±SD	Median (Range)	Mean±SD	Median (Range)				
**Lymphocyte subpopulations**	NK	15.33±5.45	16.57 (4.95-23.84)	12.97±5.73	11.91 (4.16-24.93)	63.61±8.98	64.50 (44-80)	0.17631 [1]	0.19038	0.07193	0.62599
	T CD3+	69.55±7.11	70.89 (59.02-81.68)	70.41±7.74	70.44 (57.27-84.20)	76.10±6.46	76.11 (63.34-87.66)	0.00995 [1] *	0.71523	0.00415*	0.01598*
	B CD19+	13.34±4.18	13.46 (7.11-22.15)	14.81±4.26	14.48 (8.11-22.66)	10.26±4.54	9.94 (4.35-22.12)	0.00529 [1] *	0.27644	0.03177*	0.00229*
	T CD4+CD3+	42.26±5.89	41.94 (29.91-57.94)	44.83±7.34	45.40 (33.17-63.96)	49.29±5.81	48.29 (38.97-61.22)	0.00174 [2] *	0.20358	0.00056*	0.02072*
	T CD8+CD3+	27.28±6.28	25.44 (20.06-41.76)	25.64±6.58	25.93 (16.66-38.23)	26.47±4.44	25.62 (20.58-36.71)	0.6987 [2]	0.42461	0.64022	0.64265
	CD4/CD8 ratio	1.63±0.43	1.71 (0.72-2.54)	1.90±0.75	1.69 (0.90-3.84)	1.92±0.41	1.86 (1.13-2.69)	0.1958 [2]	0.45695	0.06992	0.33692
	T CD3+CD69+ blood	4.46±2.68	3.39 (1.40-10.85)	6.54±3.25	6.64 (1.72-12.32)	3.08±1.16	2.61 (1.77-5.74)	0.00296 [2] *	0.06787	0.04375*	0.00016*
	B CD19+CD69+ blood	5.04±2.40	5.17 (1.32-9.12)	7.10±3.66	6.23 (1.77-15.42)	6.82±2.75	6.76 (2.17-15.40)	0.04961 [2] *	0.04323*	0.04987*	0.88172
	T CD3+CD25+ blood	42.26±11.34	45.44 (12.97-56.16)	47.85±16.11	45.39 (26.15-89.93)	31.85±7.25	30.02 (23.46-51.12)	0.00041 [2] *	0.21346	0.00112*	0.00051*

B CD19+CD25+ blood	30.39±9.38	30.31 (18.09-48.73)	32.01±13.88	27.49 (10-74.64)	28.64±10.91	25.28 (11.86-45.48)	0.7634 [2]	0.73526	0.67500	0.49034
CD4+CD69+ blood	5.27±2.99	4.44 (2.00-13.84)	5.41±2.86	4.84 (1.23-10.11)	3.08±1.90	2.14 (1.17-7.58)	0.00392 [2] *	0.87964	0.00333*	0.00579*
CD8+CD69+ blood	4.71±2.38	4.35 (1.06-8.85)	4.28±2.45	3.66 (1.09-10.99)	1.29±1.00	1.10 (0.29-4.19)	0.00001 [2] *	0.57918	0.00000*	0.00002*
CD4+CD25+ blood	53.48±14.22	49.49 (29.61-76.88)	59.17±17.91	56.58 (31.14-96.69)	46.82±8.60	45.10 (29.98-67.74)	0.03072 [2] *	0.27337	0.07643	0.01436*
CD8+CD25+ blood	9.50±9.28	7.25 (1.45-41.06)	8.04±7.97	5.88 (1.76-35.94)	2.02±1.03	1.76 (0.55-4.59)	0.00003 [2] *	0.57920	0.00000*	0.00001*
B CD19+PD-1	4.11±1.70	3.78 (1.89-7.18)	4.48±2.15	4.20 (1.14-10.87)	6.58±3.08	7.11 (1.73-11.34)	0.02350 [2] *	0.66512	0.00979*	0.04112*
CD4+PD-1 blood	23.26±4.47	22.13 (15.67-32.16)	19.53±5.60	17.93 (12.21-30.27)	7.74±1.66	7.80 (5.09-10.24)	0.00000 [1] *	0.02530*	0.00000*	0.00000*
CD8+PD-1 blood	19.54±7.36	17.45 (10.12-32.45)	16.77±3.65	16.50 (11.45-25.26)	4.22±2.07	4.02 (1.32-8.36)	0.00000 [2] *	0.42482	0.00000*	0.00000*
IgG VCA EBV quantitative	83.04±35.91	77.04 (31.56-148.30)	99.99±63.81	77.77 (26.22-242.62)	58.71±21.44	60.33 (20.58-89.74)	0.04617 [2] *	0.30895	0.03851*	0.05652

* statistically significant results, p <0.05; Statistically significant differences were analyzed with the use of [1]ANOVA test, [2]ANOVA Kruskal–Wallis test with post hoc tests to confirm where the differences occurred.
